# Nationwide Evaluation of an Eight-Week Interactive Online Electrocardiogram Training Program for Nurses: A Quasi-experimental Approach

**DOI:** 10.7759/cureus.94122

**Published:** 2025-10-08

**Authors:** Ankita Sharma, Ritu Rani, Vanita Kumari, Suman Dabas, Devanshi Chowdhary

**Affiliations:** 1 Nursing, All India Institute Of medical Sciences, Delhi, Delhi, IND; 2 Nursing, All India Institute of Medical Sciences, Rishikesh, Rishikesh, IND; 3 Nursing, Indian Nursing Council, Delhi, IND

**Keywords:** effectiveness, electrocardiogram, nurses, nursing, online, training

## Abstract

Background: Cardiovascular deaths are the leading cause of mortality. Early ECG diagnosis and preventive measures could prevent mortality through timely intervention. Nurses as healthcare professionals can interpret and prompt early management of life-threatening arrhythmias.

Objective: The present study aimed to provide an eight-week certificate course titled the Electrocardiogram Capacity Building Program for Nurses and assess their knowledge of ECG, arrhythmia management, and ECG interpretation skills following the learning activity.

Materials and methods: A quasi-experimental research design was selected. Eighty subjects were enrolled after a random allocation by a computer-generated randomization, and 16 hours of teaching and learning activity were carried out using an online platform. Pre-tests and post-tests were obtained using a standardized questionnaire.

Results: Significant differences were observed between pre-test and post-test scores regarding participants' (N = 76) knowledge of ECGs and their ability to identify arrhythmias. A significant improvement was noted in knowledge of the ECG from a mean pre-test score of 12.30 ± 3.51 to a post-test score of 17.42 ± 3.64. After attending the course, the number of poor performers in ECG rhythm identification dropped sharply from 36 (46.9%) to 4 (5.8%), while the number of good performers improved from 3 (4.6%) to 30 (39%).

Conclusion: No statistically significant association was found between the effectiveness of online training and demographic variables of the participants.

## Introduction

Globally, for the last 20 years, cardiovascular disorders (a type of non-communicable disorder) have been the leading cause of mortality. WHO and Global Burden of Disease estimates show cardiovascular diseases account for roughly 30-32% of all deaths (≈17-20 million deaths annually), with ischemic heart disease and stroke the most significant contributors. In India, cardiovascular diseases account for roughly one-third of deaths in recent national reports [1].

Studies have shown that rapid nurse-led ECG interpretation in the ED shortens door-to-balloon and door-to-needle times by enabling early ST-elevation myocardial infarction recognition, prompt activation of reperfusion protocols, and faster treatment, leading to improved outcomes. ECG facilities are now available in basic hospitals, and the key to preventing mortality lies in identifying abnormal rhythms and specific patterns, allowing for accurate door-to-balloon or needle time. Physicians and trained registered nurses are key to preventing cardiovascular mortality [2,3]. Although ECG analysis and interpretation are included in nursing curricula at both undergraduate and graduate levels, nurses have a poor understanding of ECG rhythm identification and interpretation [4]. Studies in India and abroad have shown that nurses have poor knowledge and competency in ECG interpretation [5-7].

In contrast, several single-center studies in India have demonstrated that ECG interpretation competency improves after training and targeted education [8,9]. Specialized short-term courses for ECG specialization are also available, allowing nurses to learn and practice ECG interpretation skills in their routine practice. ECG interpretation skills are essential for nurses working in emergency services, critical care units, and intensive care units, as they facilitate the identification and early management of life-threatening arrhythmias [10,11]. Many studies have highlighted the impact of such courses on improving patient outcomes by increasing knowledge, enhancing care quality, and facilitating better clinical decision-making among nurses [5,8,9,11].

Due to various reasons, including heavy workloads, limited learning opportunities, insufficient experience, and inadequate guidance, nurses often overlook the importance of regularly updating and practicing their ECG interpretation skills. The heart is a circulatory organ, and each year, a large proportion of patients die because of sudden cardiac arrest, either inside or outside the hospital setting. Nurses are frontline workers who come into contact with patients first; hence, a higher degree of anticipation is required, and referring these patients to the appropriate treating facility is lifesaving [4,10].

The present study aimed to conduct an eight-week certificate course titled “Electrocardiogram Capacity Building Program for Nurses” and to evaluate the effectiveness of this online training in enhancing nurses’ knowledge regarding ECG and identification of ECG rhythms, including life-threatening arrhythmias.

## Materials and methods

Study design, sample size calculation, sampling, and randomization

The study design was a one-group pre-test post quasi-experimental research design. The target population consisted of nurses across India. Based on a similar study [12], the sample size was calculated using G*Power version 3.1.9.7 (Heinrich-Heine-Universität Düsseldorf, Germany), with a two-tailed test, a 5% margin of error, a 95% confidence level, and an effect size of 0.475, resulting in a sample size of 60. After considering a 20% non-response rate, a total sample size of 72 participants was estimated. Participants were enrolled through convenience sampling using a Google link, which was conveniently shared on WhatsApp groups two weeks prior to the program's launch, and then randomly selected using computer-generated random allocation. The registration was closed once 80 participants had been randomly selected.

Inclusion criteria and materials and methods

Nurses with general nursing and midwifery qualifications or above, consenting to participate, willing to spend two hours every Friday from 5 to 7 pm for a span of eight weeks, and having a stable Internet connection for online classes were included in the study. Ethical clearance was obtained from the Institute Ethics Committee of the All India Institute of Medical Sciences (approval number: IEC-132/05.03.2021,OP-3/12.11.2021,OP-3/10.06.2025). Participants completed an online consent form before the start of the course using the pre-test questionnaire. The course was launched on June 2, 2022, and concluded on July 23, 2022. A pre-test score for the ECG Knowledge Assessment Tool (ECG-KAT) was administered at the start of the course. The program was an eight-week, interactive program conducted every Friday for a two-hour duration (16 hours of teaching and learning activities) with the assistance of Project ECHO (Extension for Community Healthcare Outcomes). Project ECHO links expert specialist teams at an academic hub with primary care clinicians in local communities, where primary care clinicians receive mentoring and feedback from specialists. To ensure adequate attendance in the session, participants were sent reminder emails and text messages every Thursday, and on the day of the program, a link for the program was sent 1/2 hour before it started. An online consent form was obtained from the participants prior to the commencement of the course. A minimum attendance of 80% was required to receive program certification. Participants with less than 80% attendance were sent text reminders to encourage them to attend the upcoming sessions.

The ECG capacity building program is composed of eight modules. A pre-test was administered to the participants 15 minutes before the commencement of the training program on June 2, 2023. Each module was provided with a two-hour duration (every Friday): the first hour and a half for learning different concepts in ECG, where subject matter experts presented their content in a well-planned manner using PowerPoint presentations (Microsoft Corp., Redmond, WA, USA), and an additional five minutes were dedicated to clearing the doubts of the participants. The last 30 minutes were allocated for two case presentations, presented by participants under the guidance of subject matter experts, with an emphasis on cardiac investigations, including ECGs, patient chest X-rays, and ECHO. At the end of the session, the subject matter expert addressed any remaining questions or concerns that the participants may have had. These activities were designed to enhance interaction and improve the learning experience. Case presentations by the participant presenters were sent to the subject matter experts one week prior to their presentation day. They were assessed by the experts, after which they were presented in the training program.

Appreciation certificates were provided to the presenters at the end of the course. The participants were provided with certificates for the "ECG Capacity Building Program" and 16 credit hours for registration renewal with the nursing council. The post-test was scheduled for the end of the eight-week program on July 23, 2023, using the same questionnaire as the pre-test.

Tool development and scoring

A self-structured tool, the ECG-KAT, was developed for pre-test and post-test assessment following a review of previous studies [5-9]. It consisted of 25 questions, which included domains (a) knowledge regarding ECG (10 questions), (b) ECG rhythm identification (10 questions), and (c) management of life-threatening arrhythmias (five questions). ECG-KAT was developed by researchers under the guidance of subject matter experts from the cardiology and nursing departments. The content validity of the tool was established by four medical and four nursing experts from different departments. The internal consistency of the tool was established using the test-retest method, yielding a Cronbach's alpha value of 0.75. A pilot study was conducted to assess the comprehensibility and feasibility of the tool. Each question carried 01 mark with no negative marking for incorrect answers. The scoring pattern for ECG-KAT was 0-10, 11-15, 15-20, and 20-25 as poor, average, good, and excellent, respectively, based on previous studies [13,14] and pilot study findings.

Statistical analysis

The data were analyzed using SPSS Statistics version 20 (IBM Corp. Released 2011. IBM SPSS Statistics for Windows, Version 20.0. Armonk, NY: IBM Corp.). Both descriptive (frequencies, percentages, mean, standard deviation, median, and range) and inferential statistics (paired t-test, one-way ANOVA) were used to interpret the data. The normality of the data was checked, and it was found to be normally distributed.

## Results

Demographic profile of participants

Out of the total selected participants of 80, four participants failed to meet the 80% attendance criterion and were therefore excluded from the final analysis. Of the 76 participants, 49 (64.5%) belonged to the 20-30 age group. The study included an almost equal distribution of males (39, 51.3%) and females (37, 48.7%). A majority of the participants, 57 (77.6%), had completed their highest qualification in B.Sc. nursing (including B.Sc. (H) nursing and post-basic B.Sc. nursing). Among them, 34 participants (44.7%) had one to five years of professional experience. Furthermore, 48 participants (63.2%) were currently employed in non-critical care units, and 62 participants (81.6%) reported that they had never attended any workshop related to ECG (Table 1).

**Table 1:** Sociodemographic profile of the participants (N = 76) G.N.M.: general nursing and midwifery, B.Sc.: bachelor of science, H: honors, M. phil: master of philosophy, ICU: intensive care unit, ECG: electrocardiogram

Demographic variables		f (%)
Age	20-30 years	49 (64.5)
	31-40 years	21 (27.6)
	41-50 years	4 (5.3)
	>50 years	2 (2.6)
Gender	Male	39 (51.3)
	Female	37 (48.7)
Highest qualification	G.N.M.	4 (5.3)
	Post-basic B.Sc. nursing	34 (44.7)
	B.Sc. (H) nursing	25 (32.9)
	M.Sc. nursing	07 (9.2)
	M. phil	06 (7.9)
Duration of work experience	Less than 1 year	09 (11.8)
	1 year to 5 years	34 (44.7)
	6 years to 10 years	18 (23.7)
	11 years to 15 years	11 (14.5)
	16 years to 20 years	03 (3.9)
	21 years to 25 years	1 (1.3)
Current department	Non-ICU	48 (63.2)
	Critical care/ICU	28 (36.8)
Attended a workshop related to ECG	No	62 (81.6)
	Yes	18.4)

Effectiveness of the ECG capacity building program

As depicted in Table 2, the number of participants with good and excellent ECG-KAT scores improved. A statistically significant improvement (p = 0.0001) with a Cohen's d of 1.06 was noted in the mean knowledge score from the pre-test to the post-test, from 12.30 ± 3.514 to 17.42 ± 3.645 (Table 3).

**Table 2:** Frequency and percentage distribution of ECG-KAT scores showing effectiveness of the ECG capacity building program on ECG knowledge (N = 76) f: number of participants, ECG-KAT: electrocardiogram knowledge assessment tool

ECG-KAT score	Pre-test f (%)	Post-test f (%)
0-10 (poor)	27 (35)	4 (5)
11-15 (average)	32 (42)	23 (30)
15-20 (good)	17 (23)	29 (38)
20-25 (excellent)	0	20 (27)

**Table 3:** Mean and standard deviation of pre-test and post-test ECG-KAT scores showing significant improvement in ECG knowledge (N = 76) # paired t-test applied, * p-value significant at <0.05 SD: standard deviation, CI: confidence interval, df: degree of freedom

ECG- KAT score	Mean (SD)	Mean difference (range)	95% CI	df	^# ^t-value (Cohen d)	*p-value
Pre-test	12.303 (3.514)	5.118 (4.819)	6.219 to 4.017	75	9.259 (1.06)	0.000*
Post-test	17.421 (3.645)					

Identification of arrhythmias

The 10 questions in the ECG-KAT consisted of identifying different arrhythmias. A specific analysis was conducted in this domain to assess the effectiveness of the course in ECG rhythm identification among participants. Scores of 0-4, 5-7, and 8-10 were considered as poor, average, and good scores, respectively. As depicted in Table 4, the number of participants with good scores in ECG rhythm identification considerably improved after attending the program.

**Table 4:** Baseline versus post intervention identification of arrhythmias (N = 76) f: number of participants, ECG: electrocardiogram

ECG rhythm identification score	Pre-test f (%)	Post-test f (%)
0-4 (poor)	36 (46.9)	4 (5.8)
5-7 (average)	37 (48.5)	42 (55.2)
8-10 (good)	3 (4.6)	30 (39)

No significant association was noted between the effectiveness of the online training program and different demographic variables (Table 5).

**Table 5:** Association between sociodemographic variables and pre-test scores of participants (N = 76) SD: standard deviation, G.N.M.: general nursing and midwifery, B.Sc. (H) nursing: bachelor of science (honors) in nursing, M.Sc. Nursing: master of science in nursing, M.Phil.: master of philosophy, Ph.D.: doctor of philosophy, ICU: intensive care unit

Demographic variable		N (76)	Mean (SD)	^#^t/F	df	*p-value
Age	20-30 years	49	12.408 (3.547)	0.303	3	0.823
	31-40 years	21	12.333 (3.786)			
	41-50 years	4	12.000 (2.309)			
	>50 years	2	10.000 (2.828)			
Gender	Male	39	12.769 (3.572)	1.192^#^	74	0.237
	Female	37	11.811 (3.430)			
Highest qualification	G.N.M.	4	12.500 (4.041)	1.841	4	0.131
	Post-basic nursing	34	11.971 (3.754)			
	B.Sc. (H) nursing	25	13.400 (3.201)			
	M.Sc. nursing	7	9.571 (1.813)			
	M.Phil/P.hD.	6	12.667 (3.445)			
Duration of education	Less than 1 year	9	12.7778 (3.153)	0.111	5	0.990
	1 year to 5 years	34	12.2059 (3.599)			
	6 years to 10 years	18	12.3889 (3.852)			
	11 years to 15 years	11	12.4545 (3.267)			
	16 years to 20 years	3	11.3333 (5.132)			
	21 years to 25 years	1	11.0000 (0)			
Current department	Non-ICU	48	11.979 (3.271)	-1.051^#^	74	0.296
	ICU/critical care	28	12.857 (3.894)			
Attending workshops	No	62	11.984 (3.395)	-1.685^#^	74	.096
	Yes	14	13.714 (3.811)			

## Discussion

The present study revealed that, after a structured ECG program, participants demonstrated a significant increase in knowledge and improved dexterity in recognizing lethal arrhythmias. A systematic review conducted by Alanezi concluded that the basic ECG training course improved nurses' knowledge, the quality of care, and patient outcomes in patients with myocardial infarction [11]. Similar results have been found when continuing education programs for ECG, such as handbooks and lecture methods, were given to nurses [7]. A comparative study of virtual versus traditional teaching methods on the interpretation of cardiac dysrhythmia in nursing students found that both methods resulted in a significant improvement in the students' interpretation of dysrhythmias. However, the virtual education group showed a 3.93-point greater improvement in mean scores than the traditional group [15]. A study conducted by Rui et al. also reported a significant gain in ECG knowledge in the group provided with the ECG instructions using a flipped classroom and lecture-based learning, which is in congruence with the present study [16]. The results of the present study, showing a significant gain in knowledge after intervention, are in congruence with those of some other studies [17,18]. The present study found no significant association between knowledge of ECG and arrhythmias and demographic variables. Similar findings were reported in a study conducted among emergency nurses in Chennai, where more than half of the nurses had moderately adequate knowledge regarding ECG skills, and no association was found between demographic variables, such as gender, educational status, and years of experience in the emergency ward [19]. Another study done in Guwahati also showed that the majority of the nurses had moderate knowledge regarding ECG [20]. In a study conducted among nursing students, it was found that 82% of the students had inadequate knowledge regarding ECG, and a significant association was also observed between the knowledge score and sociodemographic variables [21]. The findings did not align with those of our study. The effectiveness of online interactive programs can be supported by a study conducted by Shani et al. [22], which found a virtual stroke nurse certification course to be effective in improving participants’ confidence and competency in stroke care, as noted in another study by Thukral et al. [23], which assessed the efficacy of Internet-based distance learning in conjunction with local hands-on skill training in improving knowledge and skills of essential newborn care amongst in-service nursing health professionals and demonstrated that there was a significant increase in knowledge and skills scores of participants.

The strength of the present study lies in its quasi-experimental design and the use of an interactive online program for participants. The study has limitations, including the small sample size used and the post-test being taken only once at the end of the course. A more effective initiative would be to assess the retention of knowledge gained during the course by administering post-tests at regular intervals. Another limitation of the study was the inability to check the nurses in the clinical scenario in action.

Furthermore, studies can be conducted with a larger sample size, utilizing high-level fidelity simulation, and assessing knowledge in clinical settings. Additionally, various research techniques, such as randomized controlled trials and observational studies, could also be employed. The study can be replicated, and a post-test can be conducted at various intervals, such as one month or one week, to assess knowledge gain.

## Conclusions

ECG monitoring is a vital skill for nurses working in critical care areas, particularly in the prevention of sudden cardiac arrests within a hospital care setting. An understanding of abnormal and normal ECG rhythms can enable nurses to make prompt decisions in emergencies when encountering patients with life-threatening ECG rhythms. The present study supports the notion that nurses can be efficiently taught to perform ECGs and learn to identify lethal arrhythmias, thereby increasing their confidence and enabling them to make prompt decisions in emergency cases.

## Figures and Tables

**Figure 1:**
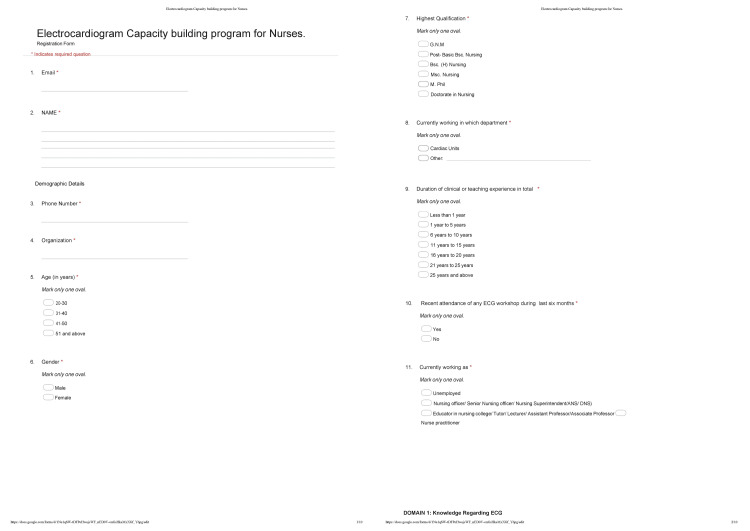
Demographic data sheet

**Figure 2:**
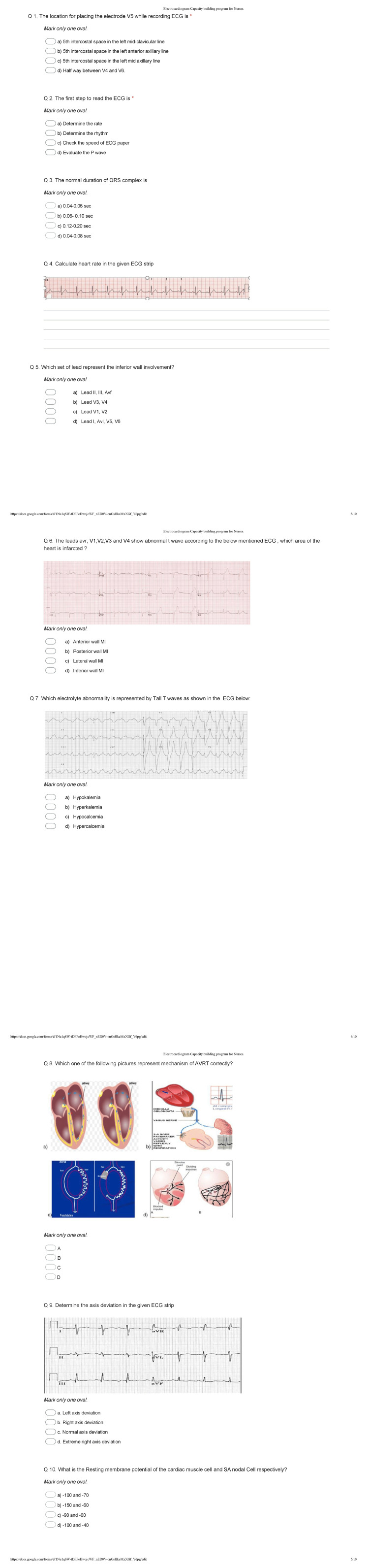
Domain 1 ECG-KAT ECG-KAT: electrocardiogram knowledge assessment tool

**Figure 3:**
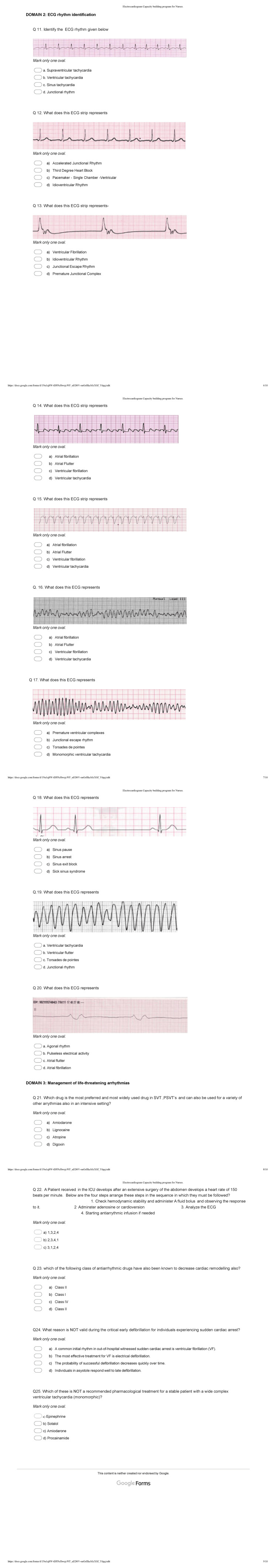
Domains 2 and 3 ECG-KAT ECG-KAT: electrocardiogram knowledge assessment tool
